# Morphologic variations of the sigmoid sinus on computed tomography: a classification-based study

**DOI:** 10.1007/s00276-026-03894-y

**Published:** 2026-05-19

**Authors:** Mahmut Sabri Medişoğlu, Melisa Öçbe

**Affiliations:** 1 Faculty of Dentistry, Department of Anatomy, Kocaeli Health and Technology University, Kocaeli, 41275 Turkey; 2 Faculty of Dentistry, Department of Oral and Maxillofacial Radiology, Kocaeli Health and Technology University, Kocaeli, 41275 Turkey

**Keywords:** Sigmoid sinus, Temporal bone CT, Anatomical variation

## Abstract

**Purpose:**

The sigmoid sinus is a vital dural venous structure whose anatomical variability has direct implications for lateral skull base and otologic surgeries. Understanding the sinus’s positional variations is considered important for minimizing surgical risks. This study aimed to evaluate the morphological classification of the sigmoid sinus using a surgical reference-based system and to assess its relationship with demographic variables.

**Methods:**

A retrospective analysis was conducted on high-resolution temporal bone CT scans of 241 patients (114 females [47.30%], 127 males [52.70%]) with an age range of 18–92 years (mean age: 47.95 ± 17.84). The morphology of the sigmoid sinus was classified into four types based on the system defined by Dong-Il Sun et al., using three anatomical reference lines. The width and depth of the sigmoid sinus were measured bilaterally and analyzed in relation to age and gender. Statistical analysis included paired tests for bilateral measurements, chi-square tests and ordinal logistic regression for associations with age and sex, and reliability assessment using Cohen’s kappa and intraclass correlation coefficients.

**Results:**

A total of 241 patients were included. Mean sigmoid sinus depth and width were 6.57 ± 1.73 mm and 14.55 ± 4.06 mm on the right, and 6.33 ± 1.52 mm and 15.18 ± 4.93 mm on the left, respectively. Left-sided width was significantly greater than the right (*p* < 0.05), whereas depth did not differ. Type 3 and Type 4 configurations predominated bilaterally; Type 1 was not observed. Age was significantly associated with left-sided sigmoid sinus type (*p* = 0.0048), with more medial configurations in older individuals. Ordinal regression showed decreasing odds of higher-type anatomy with increasing age (OR = 0.84 per decade). Gender was associated with left-sided type distribution but not independently predictive after adjustment. Inter- and intraobserver reliability was excellent.

**Conclusion:**

The positional morphology of the sigmoid sinus varies significantly with age and gender. The absence of Type 1 and predominance of high-risk configurations in younger individuals suggests the potential relevance of individualized preoperative assessment. This classification system may provide additional anatomical insight for radiologic evaluation; however, its direct impact on surgical decision-making requires further validation.

## Introduction

The sigmoid sinus is a major venous structure of the posterior cranial fossa [1–4] and represents a critical anatomical landmark during otologic and lateral skull base surgery. Its position relative to surrounding osseous structures influences surgical exposure, drilling trajectories, and the risk of vascular injury [4–7]. Variations in the course and prominence of the sigmoid sinus are therefore of particular importance for preoperative imaging assessment and surgical planning [8–14].

Several classification systems have been proposed to describe sigmoid sinus position based on reproducible anatomical reference lines [14, 15], among which the system introduced by Dong-Il Sun et al. has gained attention for its practical, surgery-oriented approach [16]. This classification categorizes the sigmoid sinus according to its anterolateral extension within the temporal bone, allowing standardized description across imaging studies [16–18]. Despite its potential clinical relevance, data on the distribution of sigmoid sinus types in adult populations remain limited, and reported frequencies vary across cadaveric and radiologic series.

In addition, the anatomical factors influencing sigmoid sinus position are not fully understood. Temporal bone morphology is known to undergo age-related remodeling, and mastoid pneumatization has been shown to affect the spatial relationships between the sigmoid sinus and adjacent structures [9–11]. Sex-related differences in cranial and temporal bone morphology have also been reported, although their influence on sigmoid sinus configuration remains inconsistent across studies. Nevertheless, few investigations have systematically evaluated age- and sex-related patterns of sigmoid sinus types using computed tomography, and bilateral assessments within the same individuals are particularly scarce [16–19]. Beyond classification frequency, the relationship between sigmoid sinus type and morphometric parameters such as sinus width and depth has received limited attention. Quantitative evaluation of these dimensions may provide additional insight into how positional categories relate to underlying anatomical variation and may improve radiologic communication for surgical planning.

Therefore, the aim of the present study was to evaluate the distribution of sigmoid sinus types using the Dong-Il Sun classification on computed tomography (CT) [16], to assess bilateral patterns, and to analyze their associations with age, gender, and morphometric measurements of sinus width and depth.

## Materials and methods

### Ethical considerations

This retrospective research adhered to established ethical standards and received approval from the Non-Invasive Clinical Research Ethics Committee of Kocaeli Health and Technology University (Approval No: 2025-178). The study protocol was assessed and authorized by the Institutional Review Board (IRB), confirming its alignment with ethical guidelines. All procedures were conducted in accordance with the principles outlined in the Declaration of Helsinki (1964) and its subsequent revisions. As this study has a retrospective design, the requirement for informed consent was waived because all data were anonymized and analyzed retrospectively.

### Study group selection

This retrospective study involved the analysis of Computed Tomography (CT) scans with a focus on the sigmoid sinus. Imaging data were retrieved from the Department of Radiology at Kocaeli City Hospital between 2022 and February 2025. The CT scans were originally acquired for various diagnostic and therapeutic purposes unrelated to the study, such as headache evaluation, trauma, sinus pathology, or preoperative assessments. Cases with a history of temporal bone surgery, congenital craniofacial anomalies, destructive temporal bone lesions, or imaging findings that precluded reliable assessment of sigmoid sinus anatomy were excluded. Because of the retrospective design, detailed otologic diagnoses such as chronic otitis media or inflammatory middle ear disease were not consistently available in the medical records and therefore could not be analyzed as independent variables. Although no cases with documented chronic middle ear disease were identified based on clinical records and ICD coding, the possibility of subclinical or undiagnosed conditions cannot be entirely excluded due to the retrospective design. No patient had undergone prior otologic or skull base surgery that could directly alter sigmoid sinus position. All CT examinations were reviewed solely for anatomical evaluation.

The study focused on morphologic assessment of the sigmoid sinus using a standardized CT-based classification system, independent of clinical indication. Patient demographic variables (age and sex) were recorded to explore potential associations with sigmoid sinus configuration. All measurements and classifications were performed on non-contrast computed tomography (CT) images using bone window settings. Image analysis was conducted on a dedicated radiologic workstation that allowed synchronized multiplanar reconstruction (axial, coronal, and sagittal views).

Inclusion criteria were as follows; patients aged 18 years or older, availability of high-resolution cranial or temporal bone CT scans encompassing the posterior cranial fossa, optimal image quality with clear visualization of the sigmoid sinus and adjacent anatomical landmarks, no evidence of extensive metallic artifacts or motion blurring that could compromise the assessment of vascular structures, normal anatomical continuity of the sigmoid sinus, allowing for bilateral evaluation. Exclusion criteria included patients with known or suspected intracranial pathology (e.g., tumors, vascular malformations, thrombosis) affecting the sigmoid sinus or adjacent venous sinuses, history of cranial or otologic surgery, including mastoidectomy, shunt placement, or craniotomy, presence of space-occupying lesions, fractures, or congenital anomalies in the posterior cranial fossa, emergency imaging cases where image quality or anatomical visibility was inadequate CT scans limited to the facial skeleton or not extending to the region of the sigmoid sinus.

### Imaging parameters

All CT examinations were performed using multidetector scanners (Revolution™ EVO or Optima™ CT660; GE Healthcare, Tokyo, Japan) with standardized acquisition protocols. Images were obtained using a bone reconstruction algorithm with thin-section axial slices suitable for detailed temporal bone evaluation.All included scans demonstrated optimal diagnostic quality, free from motion or metallic artifacts. Image analysis was carried out using multiplanar reformation (MPR) in axial, coronal, and sagittal planes. A certified Oral and Maxillofacial Radiologist (XX) and an Anatomy Specialist (XXX), each with experience in head and neck imaging, independently evaluated all scans while blinded to patient demographic information. Assessments were conducted in a standardized radiologic viewing environment, allowing synchronized visualization of the three planes in bone window settings to ensure consistency and diagnostic accuracy. Due to slice orientation, the common crus may not be visualized as a single unified structure on all axial images; therefore, the reference level was defined based on the recognizable junctional region of the superior and posterior semicircular canals. Intraobserver reliability was measured with a subset, after 3 months from the first evaluation by the observer 1 (XX).

### Classification of sigmoid sinus variations

For each side, the entire axial image stack encompassing the temporal bone was systematically reviewed to identify the level at which the sigmoid sinus demonstrated its maximum lateral or anterior protrusion into the mastoid region. This slice was selected individually for each side and served as the reference level for both classification and morphometric measurements.

All axial slices of the temporal bone were reviewed in the bone algorithm to identify the level at which the sigmoid sinus contour was most anteriorly protrusive. The measurement slice was defined as the axial image showing the maximum anterior convexity of the sigmoid sinus groove (sigmoid sulcus) relative to the posterior temporal bone. Measurements were obtained on this slice for each side. If adjacent slices showed comparable protrusion, the slice with the largest measured anteroposterior distance was selected. Classification reference lines were drawn on a standardized axial level defined by the posterior semicircular canal (PSCC) common crus level, identified as the first axial slice in which the PSCC common crus is clearly visualized bilaterally. Reference lines were drawn using the same axial level for both sides in each patient to ensure within-case consistency.

**Fig. 1:**
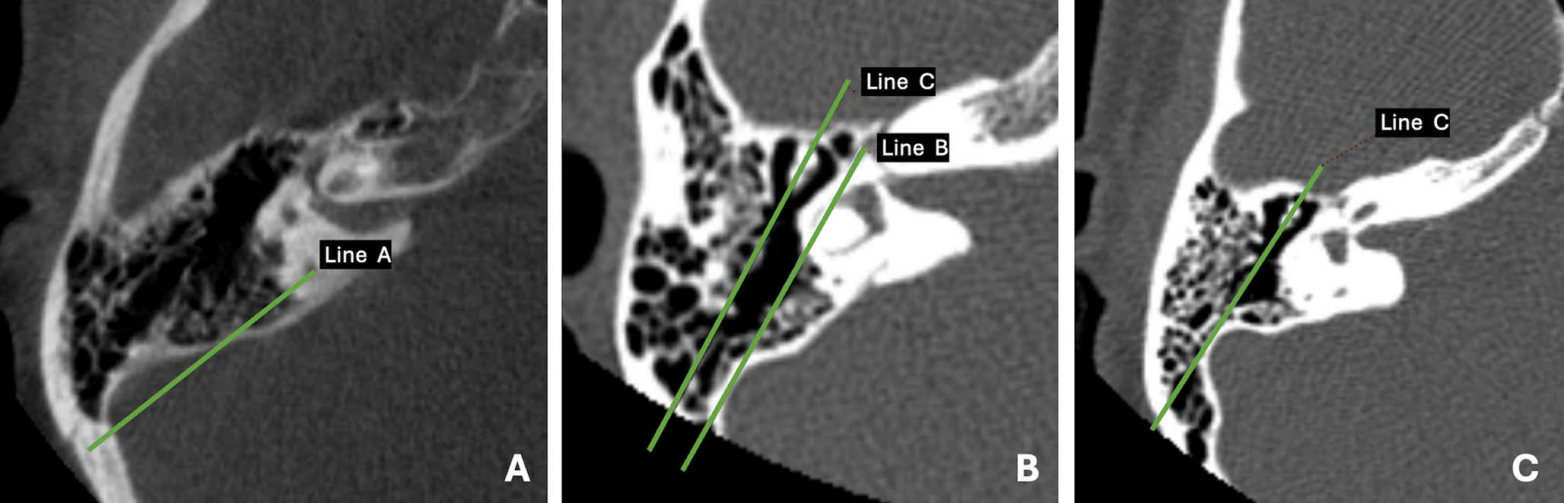
Axial CT images illustrating the anterior-lateral positioning of sigmoid sinus classification according to Dong-Il Sun et al. Reference level corresponding to the common crus region (junction of the superior and posterior semicircular canals), which may appear partially depending on axial slice orientation. (A–C) are used to define sinus position: **A** is showing Line A, **B** showing Lines B and C, and **C** presenting Line C

Morphological classification of the sigmoid sinus was performed using the system described by Dong-Il Sun et al., which evaluates the prominence and surgical accessibility of the sinus based on its position relative to three anatomical reference lines drawn on axial CT images [16]. These reference lines were defined as follows:

*Line 1:* A posterior reference line drawn at the axial level corresponding to the common crus region, defined as the junction of the superior and posterior semicircular canals. This level was identified based on the recognizable convergence of these canals on axial CT images; however, due to slice orientation, the common crus may not always be visualized as a single unified structure. (Fig. 1A).

*Line 2:* A posterior line projected along the tympanic segment of the facial nerve, representing the surgical route for middle ear access and tympanotomy. (Fig. 1B).

*Line 3:* A posterior line drawn along the malleal-incudal (malleus-incus) axis, corresponding to the superior middle ear or attic access (epitympanotomy) (Fig. 1C).

All measurements were performed on non-contrast CT in the bone window and were based on the bony sigmoid sulcus (groove) rather than the venous lumen. The measurement boundary was defined by the cortical margins of the sigmoid sulcus visible on the bone algorithm reconstruction. All reference lines were constructed parallel to the posterior cortical margin of the temporal bone on the same axial plane. Apparent deviations in the figures may be related to anatomical curvature, slice angulation, or 2D projection of 3D structures.

**Fig. 2:**
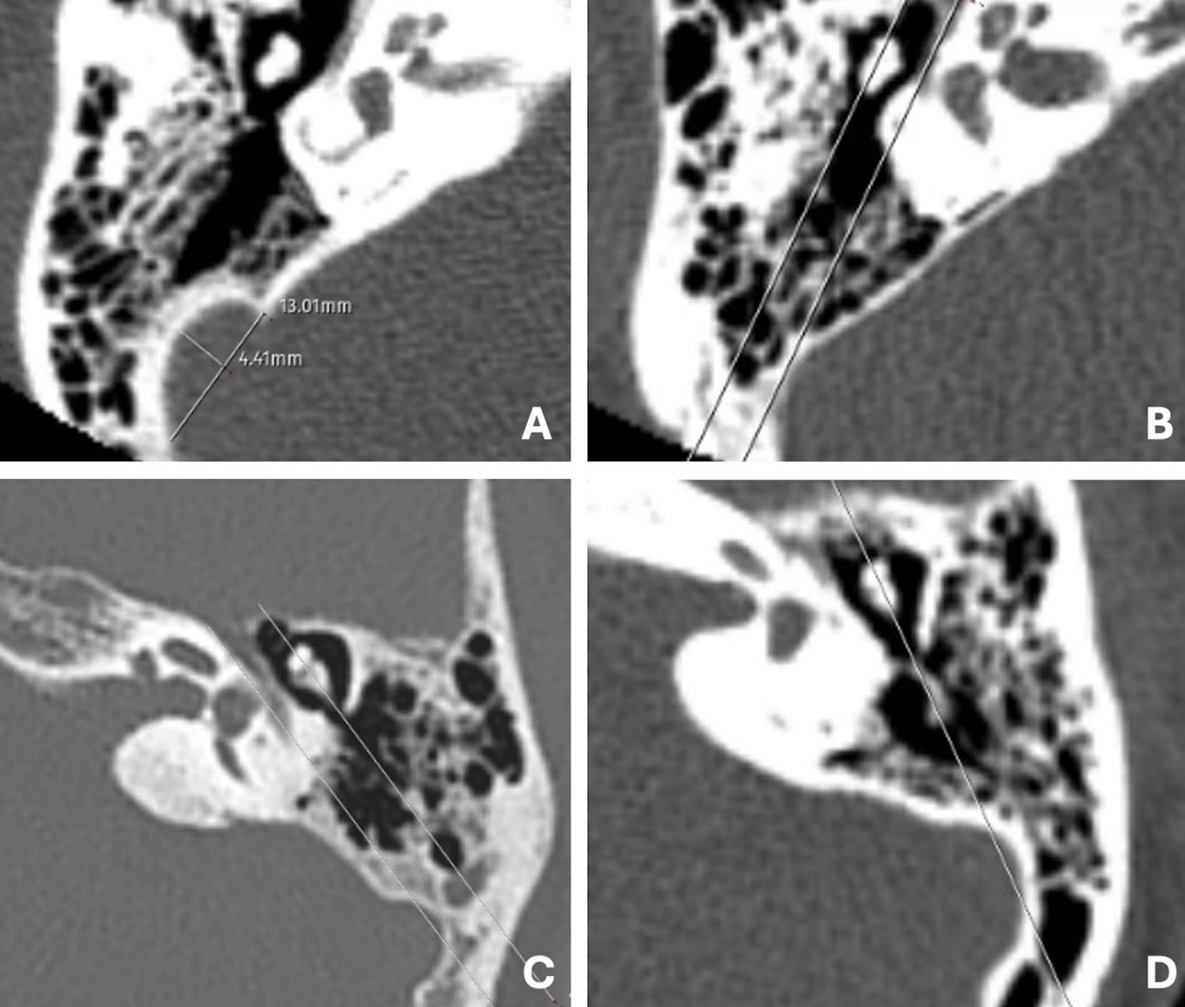
**A** Axial CT image illustrating morphometric evaluation of a left sigmoid sinus classified as Type 2, including measurements of width and depth. Depth is defined as the maximum anteroposterior distance from the posterior temporal bone to the most anterior convex point of the sigmoid sinus, while width is defined as the maximum mediolateral distance between the medial and lateral sinus margins. **B** Representative example of sigmoid sinus Type 2. **C** Representative example of sigmoid sinus Type 3. **D** Representative example of sigmoid sinus Type 4

For quantitative morphometric measurements of the sigmoid sinus were performed to assess its anatomical dimensions. For each patient, the width and depth of the sigmoid sinus were measured bilaterally on axial CT images in the bone window setting:

*Sigmoid Sinus Width:* Defined as the maximum mediolateral distance between the lateral dural margin of the sinus and the medial bony boundary adjacent to the cerebellar surface. Measurements were performed at the level where the sinus reached its greatest horizontal span within the mastoid portion (Fig. 2A).

*Sigmoid Sinus Depth:* Defined as the maximum anteroposterior distance from the posterior surface of the temporal bone to the most anterior convex point of the sigmoid sinus wall, measured on the same axial slice as the width. This represents how deeply the sinus protrudes into the mastoid cavity (Fig. 2A).

All measurements were performed on bone reconstruction images using a consistent bone window setting (institutional bone preset). Digital calipers were used, and measurements were recorded in millimeters. Based on the location of the most protruding point of the sigmoid sinus in relation to these reference lines, each sinus was categorized into one of four types:

*Type 1:* The most protruding portion of the sigmoid sinus lies medial or posterior to Line 1. This type indicates a low surgical risk and favorable access to the mastoid and middle ear.

Type 2: The sinus protrudes between Line 1 and Line 2, representing a moderate risk and requiring careful surgical approach during middle ear procedures (Fig. 2B).

*Type 3:* The most prominent portion of the sinus is located between Line 2 and Line 3, signifying a classification-based higher risk due to reduced access and proximity to critical structures (Fig. 2C).

*Type 4:* The sinus extends lateral or anterior to Line 3, posing a classification-based higher risk and presenting significant limitations for operative access (Fig. 2D).

All measurements and classifications were performed bilaterally on axial bone window CT sections. All measurements were recorded in millimeters (mm) using the measurement tools provided by the dedicated CT workstation.

### Statistical analysis

All statistical analyses were performed using IBM SPSS Statistics (Version 24.0; IBM Corp., Armonk, NY, USA). Descriptive statistics were calculated for demographic and morphometric variables. Continuous variables were summarized as mean ± standard deviation (SD), minimum, and maximum values, whereas categorical variables were expressed as frequencies and percentages.

Normality of continuous variables (sigmoid sinus width and depth) was assessed using the Shapiro–Wilk test and visual inspection of histograms. Because measurements were obtained bilaterally from the same individuals, right–left comparisons were treated as paired data. Accordingly, paired-samples t tests were used for normally distributed variables, and the Wilcoxon signed-rank test was applied when normality assumptions were not met. Mean differences with 95% confidence intervals (CIs) were reported to quantify effect size.

Sigmoid sinus type, classified as an ordinal categorical variable (Types 2–4), was analyzed using non-parametric methods. Associations between sigmoid sinus type and categorical variables (sex and age group) were assessed using the chi-square test of independence. Effect size was quantified using Cramér’s V. Age was additionally explored as a continuous variable in exploratory analyses using ordinal regression models to assess its association with sigmoid sinus type, acknowledging the ordinal nature of the outcome variable. Right and left sides were analyzed separately because both observations originate from the same individual and therefore cannot be considered independent.

Because multiple hypothesis tests were performed, all statistical analyses were considered exploratory. Accordingly, p-values were interpreted cautiously, and emphasis was placed on effect sizes and confidence intervals rather than statistical significance alone. A two-sided p value < 0.05 was considered statistically significant.

Interobserver reliability was assessed to evaluate measurement reproducibility. Agreement for categorical classification of sigmoid sinus type was evaluated using Cohen’s kappa (κ). Reliability of continuous measurements (sigmoid sinus width and depth) was assessed using the intraclass correlation coefficient (ICC), based on a two-way random-effects model with absolute agreement. ICC values were interpreted according to commonly accepted thresholds (poor < 0.50, moderate 0.50–0.75, good 0.75–0.90, excellent > 0.90). To assess intra-observer reliability, a subset of the dataset was re-evaluated by the same observer after a washout period of three months. The observer repeated both the categorical classification of sigmoid sinus type and the quantitative morphometric measurements (sigmoid sinus width and depth) using the same measurement protocol and imaging conditions as in the initial assessment. Intra-observer agreement for categorical classification was evaluated using Cohen’s kappa (κ). Reliability for continuous measurements (sigmoid sinus width and depth) was assessed using the intraclass correlation coefficient (ICC) based on a two-way random-effects model with absolute agreement. ICC values were interpreted as follows: < 0.50 poor, 0.50–0.75 moderate, 0.75–0.90 good, and > 0.90 excellent reliability. All analyses were performed using standard statistical software.

All statistical analyses were conducted in accordance with recommended reporting standards for observational imaging studies.

## Results

### Demographic and morphometric characteristics

A total of 241 patients were included in this study, consisting of 114 females (47.30%) and 127 males (52.70%). The mean age was 47.95 ± 17.84 years, with participants ranging in age from 18 to 92 years. The distribution of participants by age group was as follows: 19–30 years (n = 40), 31–45 years (n = 72), 46–60 years (n = 67), and 61 + years (n = 62) (Table 1).

**Table 1:** Distribution of the participants

Parameter	Value
Total participants (n)	241
Female, n (%)	114 (47.3%)
Male, n (%)	127 (52.7%)
Mean age (years)	47.95 ± 17.84
Age range (years)	18–92

Values are presented as mean ± standard deviation or number (%)

The mean right sigmoid sinus depth was 6.57 ± 1.73 mm, with a range from 2.54 to 10.79 mm, while the right sigmoid sinus width averaged 14.55 ± 4.06 mm, ranging between 8.27 and 23.65 mm. For the left sigmoid sinus, the mean depth was 6.33 ± 1.52 mm (range: 3.06 to 10.08 mm) and the mean width was 15.18 ± 4.93 mm (range: 7.32 to 29.36 mm) (Table 2).

**Table 2:** Sigmoid sinus measurements (mm)

Measurement	Mean ± SD	Min–Max
Right sigmoid depth	6.57 ± 1.73	2.54–10.79
Left sigmoid depth	6.33 ± 1.52	3.06–10.08
Right sigmoid width	14.55 ± 4.06	8.27–23.65
Left sigmoid width	15.18 ± 4.93	7.32–29.36

Right–left comparisons were performed using paired statistical tests because both measurements were obtained from the same individual

Because right–left measurements were obtained from the same individuals, paired comparisons were performed using the Wilcoxon signed-rank test. Left width was significantly greater than right width (mean difference: 0.63 mm, 95% CI 0.12 to 1.14; *p* = 0.00039; effect size r = 0.23). In contrast, depth did not differ significantly between sides (mean difference: − 0.25 mm, 95% CI − 0.52 to 0.03; *p* = 0.132; r = 0.10) (Table 3).

**Table 3:** Paired comparison (right vs. left)

Variable	Mean difference (L–R)	95% CI	*p* value	Effect size (r)
Depth	− 0.25 mm	− 0.52 to 0.03	0.132	0.10
Width	+ 0.63 mm	0.12 to 1.14	0.00039	0.23

Paired t-tests or Wilcoxon signed-rank tests were applied depending on data distribution. Effect sizes are reported as r. A p value < 0.05 was considered statistically significant

### Distribution of sigmoid sinus types

No cases were classified as Type 1 on either side. On the right, Type 2 was observed in 35 cases (14.5%), Type 3 in 99 cases (41.1%), and Type 4 in 107 cases (44.4%). On the left, Type 2 was observed in 36 cases (14.9%), Type 3 in 114 cases (47.3%), and Type 4 in 91 cases (37.8%) (Table 4).

**Table 4:** Distribution of sigmoid sinus types by side

Type	Right n (%)	Left n (%)
Type 1	0 (0.0)	0 (0.0)
Type 2	35 (14.5)	36 (14.9)
Type 3	99 (41.1)	114 (47.3)
Type 4	107 (44.4)	91 (37.8)
**Total**	**241 (100)**	**241 (100)**

Values are expressed as number (%). Sigmoid sinus types were classified according to the Dong-Il Sun et al. system. Type 1 was not observed in this cohort. Right and left sides were analyzed separately due to within-subject dependence. Percentages are calculated within each side

When morphometric parameters were compared across sigmoid sinus types, significant differences were observed on the right side. Kruskal–Wallis analysis demonstrated that right sigmoid sinus width differed significantly among Types 2–4 (H = 9.00, *p* = 0.011), as did right sigmoid sinus depth (H = 25.39, *p* < 0.001). Median values indicated larger width and depth measurements in Type 2 compared with Types 3 and 4.

On the left side, no statistically significant differences were observed across sigmoid sinus types for either width (H = 3.19, *p* = 0.203) or depth (H = 3.83, *p* = 0.147). These findings suggest that morphometric variation across classification types is more pronounced on the right side and should be interpreted cautiously given the paired nature of the data.

### Right–left concordance of sigmoid sinus type

Right–left type concordance (same type on both sides) was present in 158/241 patients (65.6%). The most frequent concordant patterns were Type 3/Type 3 (n = 67) and Type 4/Type 4 (n = 68), followed by Type 2/Type 2 (n = 23). Among discordant cases, Type 4 on one side with Type 3 on the other was the most common asymmetry (right Type 4/left Type 3: n = 35; right Type 3/left Type 4: n = 23).

### Age and sigmoid sinus type

Age was evaluated using predefined age groups (18–30, 31–45, 46–60, 61 +) and compared with sigmoid sinus type using chi-square tests, with effect size reported as Cramér’s V. For the right sigmoid sinus, the age-group association did not reach statistical significance (χ^2^(6) = 11.31, *p* = 0.079; Cramér’s V = 0.153). For the left sigmoid sinus, there was a significant association between age group and type (χ^2^(6) = 18.66, *p* = 0.0048; Cramér’s V = 0.197). On the left side, Type 4 was most prevalent in the 18–30 group (48.9%), whereas Type 2 became more frequent in older groups (e.g., 20.3% in 46–60 and 23.6% in 61 +), indicating a trend toward more medially positioned configurations with increasing age.

To further explore age as a continuous predictor while respecting the ordinal nature of the outcome (Types 2–4), ordinal logistic regression models were fitted separately for each side (exploratory analysis). Increasing age was associated with lower odds of a more laterally/anteriorly positioned type on both sides: per 10-year increase, the odds of being in a higher type category decreased for the right (OR 0.84, 95% CI 0.73–0.96; *p* = 0.011) and the left (OR 0.84, 95% CI 0.73–0.97; *p* = 0.015) (Table 5).

**Table 5:** Distribution of sigmoid sinus types by age group

Age group (years)	Right			Left		
	Type 2 n (%)	Type 3 n (%)	Type 4 n (%)	Type 2 n (%)	Type 3 n (%)	Type 4 n (%)
18–30 (n = 40)	4 (10.0)	16 (40.0)	20 (50.0)	8 (20.0)	15 (37.5)	17 (42.5)
31–45 (n = 72)	5 (6.9)	34 (47.2)	33 (45.8)	2 (2.8)	40 (55.6)	30 (41.7)
46–60 (n = 67)	10 (14.9)	25 (37.3)	32 (47.8)	10 (14.9)	31 (46.3)	26 (38.8)
≥ 61 (n = 62)	16 (25.8)	24 (38.7)	22 (35.5)	16 (25.8)	28 (45.2)	18 (29.0)

Values are presented as number (%). Associations between age group and sigmoid sinus type were evaluated using the chi-square test. Effect size was calculated using Cramér’s V. Because multiple comparisons were performed, results were interpreted in an exploratory manner. Right and left sides were analyzed separately due to within-subject correlation. Right side: χ^2^(6) = 11.31, *p* = 0.079, Cramér’s V = 0.153; Left side: χ^2^(6) = 18.66, *p* = 0.0048, Cramér’s V = 0.197

### Gender and sigmoid sinus type

Sex differences in sigmoid sinus type were assessed using chi-square tests with Cramér’s V. For the right sigmoid sinus, no significant association with sex was observed (χ^2^(2) = 1.85, *p* = 0.396; Cramér’s V = 0.088). For the left sigmoid sinus, a significant association with sex was identified (χ^2^(2) = 10.79, *p* = 0.0045; Cramér’s V = 0.212). On the left side, females showed a higher proportion of Type 3 (57.9%) compared with males (37.8%), whereas males had a higher proportion of Type 2 (19.7%) and Type 4 (42.5%) relative to females (9.6% and 32.5%, respectively). In exploratory ordinal regression adjusting for age, sex was not an independent predictor of sigmoid sinus type (male vs female: right OR 1.06, 95% CI 0.66–1.73; *p* = 0.801; left OR 1.00, 95% CI 0.62–1.63; *p* = 0.992) (Table 6).

**Table 6:** Distribution of sigmoid sinus types by gender

Sex	Right			Left		
	Type 2 n (%)	Type 3 n (%)	Type 4 n (%)	Type 2 n (%)	Type 3 n (%)	Type 4 n (%)
Female (n = 114)	15 (13.2)	52 (45.6)	47 (41.2)	11 (9.6)	66 (57.9)	37 (32.5)
Male (n = 127)	20 (15.7)	47 (37.0)	60 (47.2)	25 (19.7)	48 (37.8)	54 (42.5)

Values are presented as number (%). Associations between sex and sigmoid sinus type were assessed using the chi-square test with Cramér’s V as a measure of effect size. Right and left sides were analyzed separately. Percentages represent proportions within each sex category. Right side: χ^2^(2) = 1.85, *p* = 0.396, Cramér’s V = 0.088; Left side: χ^2^(2) = 10.79, *p* = 0.0045, Cramér’s V = 0.212

### Inter- and intraobserver reliability

Interobserver agreement for categorical classification of sigmoid sinus type was assessed using Cohen’s kappa. Agreement was excellent for the right side (κ = 0.87) and moderate for the left side (κ = 0.59). Reliability of continuous morphometric measurements was assessed using the intraclass correlation coefficient (ICC) based on a two-way random-effects model with absolute agreement. Interobserver reliability was excellent for all measurements, with ICC values of 0.99 for right sigmoid sinus depth, 0.92 for left sigmoid sinus depth, 1.00 for right sigmoid sinus width, and 0.999 for left sigmoid sinus width. These findings indicate very high reproducibility of the measurement protocol.

Intra-observer agreement for sigmoid sinus type classification was excellent on the right side (κ = 0.82) and good to excellent on the left side (κ = 0.75), indicating high consistency of categorical assessments over time. For continuous morphometric measurements, intra-observer reliability was excellent for all parameters. The ICC for right sigmoid sinus depth was 0.99, and for left depth was 0.96. Similarly, the ICC for right sigmoid sinus width was 0.98, and for left width was 0.998, demonstrating very high repeatability of linear measurements across repeated evaluations.

## Discussion

The sigmoid sinus not only demonstrates considerable positional variability but also exhibits complex relationships with adjacent temporal bone structures, posing challenges during neurosurgical procedures. In lateral skull base surgery, understanding of the anatomical relationships among the sigmoid sinus, jugular bulb, external auditory canal, and facial nerve is considered important for minimizing potential complications. Although the clinical variability of the sigmoid sinus is well acknowledged, precise knowledge regarding the extent and patterns of this variability remains limited. The relationship between mastoid pneumatization and sigmoid sinus variations continues to be a subject of debate [16, 17]. Some reports suggest that the position of surgically significant structures, including the sigmoid sinus, is seldom influenced by sclerotic changes in the middle and inner ear [17]. The present study provides a CT-based evaluation of sigmoid sinus morphology using a surgical reference–based classification system, with bilateral distribution, morphometric characteristics, and associations with age and sex.

Variations in sigmoid sinus shape and position may influence surgical access to the tympanic cavity, mastoid antrum, and membranous labyrinth [18–20]. As the sigmoid sinus represents a critical landmark during otologic and neurotologic procedures, its position influences surgical exposure, working corridors, and procedural safety. Variations in its course relative to the posterior semicircular canal, jugular bulb, and surrounding temporal bone structures may complicate access during mastoidectomy or presigmoid approaches. For this reason, several classification systems have been proposed to describe sigmoid sinus position using reproducible anatomical reference lines.

Several anatomical and surgical classification systems have been proposed to describe variability in the position of the sigmoid sinus and its relationship to surrounding temporal bone structures. Early morphometric work by Oppel and Mulch reported that the average dimensions of Trautmann’s triangle are approximately 1.2 × 0.8 cm, emphasizing its limited and variable surgical corridor [20]. Subsequent studies have demonstrated substantial interindividual variability; for example, Nitek et al. reported a mean surface area of 175.9 mm^2^ (range: 84–356 mm^2^), which shows marked differences in sigmoid sinus course and jugular fossa depth [21]. From a surgical perspective, Friedman and Lee revealed that anterior displacement of the sigmoid sinus may restrict access to the internal auditory canal during translabyrinthine approaches, underscoring the clinical relevance of positional variability [22, 23].

Similary, various classification systems have been introduced to categorize sigmoid sinus morphology. Ichijo et al. proposed a morphology-based classification focusing on sinus shape and spatial relationships [15]. Kayalıoğlu et al. subsequently described five anatomical types based on the entire course of the sigmoid sinus within the temporal bone, showing its three-dimensional trajectory [24]. Sarmiento et al. introduced a surgically oriented classification using cadaveric specimens, defining three types according to the relationship between the sigmoid sinus and Trautmann’s triangle: Type 1, in which the sinus is located posteriorly with a wide triangle; Type 2, characterized by anterior displacement and partial narrowing of the triangle; and Type 3, in which medial displacement results in a markedly reduced surgical window [14]. Other systems have focused not on sinus position itself but on mastoid pneumatization. Han et al., for example, classified temporal bones into four groups according to mastoid air cell volume, ranging from poorly to extensively pneumatized (mean volumes 12.41–20.73 cm^3^) [25]. Although frequently cited in discussions of sigmoid sinus anatomy, this classification primarily reflects pneumatization rather than direct sinus location. In the present study, we adopted the classification proposed by Dong-Il Sun et al., which evaluates the prominence and surgical accessibility of the sigmoid sinus using three reproducible anatomical reference lines on CT imaging [16] (Table 7). This system combines spatial relationships relevant to surgical exposure and allows standardized assessment across patients, making it particularly suitable for radiologic analysis and comparison of bilateral anatomy.

**Table 7:** Summary of major classification systems and anatomical concepts related to sigmoid sinus position

Author (Refs.)	Basis of classification	Key parameters	Main focus	Clinical relevance
Oppel & Mulch [20]	Morphometric measurement	Size of Trautmann’s triangle	Surgical corridor size	Shows limited and variable access window
Nitek et al. [21]	Quantitative morphometry	Surface area of Trautmann’s triangle	Variability of sinus course and jugular fossa depth	Shows interindividual anatomical variability
Friedman [22], Lee [23]	Surgical observation	Anterior displacement of sigmoid sinus	Translabyrinthine access	Anterior sinus limits surgical exposure
Ichijo et al. [15]	Morphologic classification	Sinus shape and spatial configuration	Descriptive anatomy	Categorizes sigmoid sinus morphology
Kayalıoğlu et al. [24]	Anatomical course-based classification	Entire sigmoid sinus trajectory	Structural anatomy	Defines five anatomical variants
Sarmiento et al. [14]	Surgical classification (cadaveric)	Relation to Trautmann’s triangle	Surgical accessibility	Type 1–3 classification
Han et al. [25]	Pneumatization-based classification	Mastoid air cell volume	Degree of pneumatization	Indirect effect on sinus position
Dong-Il Sun et al. [16]	Reference-line–based classification	Three anatomical reference lines	Surgical accessibility & prominence	Radiologic, reproducible, clinically oriented

This study showed the absence of Type 1 sigmoid sinus configuration in both right and left sides. Previous cadaveric and radiologic studies have reported Type 1 as an uncommon but identifiable variant [17, 18, 25, 26]. The absence of this type in our cohort may reflect differences in population characteristics, or classification thresholds, but may also be related to the degree of mastoid air cell development. Increased mastoid pneumatization has been shown to influence the anterior and lateral displacement of the sigmoid sinus, potentially reducing the likelihood of configurations corresponding to Type 1. Because the present study did not directly quantify mastoid pneumatization, this interpretation should be considered speculative. Nevertheless, the consistent lack of Type 1 across both sides suggests that pneumatization-related anatomical remodeling may play an important role in shaping sigmoid sinus position in adult populations. Future studies including direct measurements of mastoid air cell volume may help clarify this relationship.

In the present study, age demonstrated a significant association with sigmoid sinus type, particularly on the left side, with older individuals showing a higher frequency of more medially positioned configurations. This finding is consistent with previous anatomical and radiological literature suggesting that temporal bone morphology continues to remodel throughout adulthood, influenced by pneumatization patterns and bone remodeling processes [17, 18, 25, 27, 28]. Several studies have shown that mastoid pneumatization and surrounding bony structures may undergo progressive changes over time, potentially altering the spatial relationship between the sigmoid sinus and adjacent anatomical landmarks [17, 25, 27, 28]. As increased pneumatization has been found to be associated with anterior and lateral displacement of the sigmoid sinus, age-related differences in pneumatization may partially explain the observed distribution of sigmoid sinus types in the present cohort. In addition, this study showed a higher prevalence of Type 4 configuration in younger adults, whereas more medially positioned types were more frequent with increasing age. From a clinical perspective, these findings may have practical implications for preoperative planning, although their applicability remains uncertain. Higher-type sigmoid sinus configurations (Types 3–4), which reflect a more anterior or lateral position, may influence surgical exposure during otologic or lateral skull base procedures. Therefore, reporting sigmoid sinus type—particularly Types 3 and 4—within radiologic assessments may contribute to improved anatomical understanding in radiologic evaluation. Nevertheless, such suggestions remain hypothesis-generating and should be validated in future studies integrating operative outcomes and volumetric measures of mastoid pneumatization.

Gender-related differences were also observed in univariate analyses, particularly on the left side; however, sex did not remain an independent predictor after adjustment for age in ordinal regression models. This finding aligns with prior reports indicating that while sexual dimorphism may influence temporal bone size or pneumatization patterns, its effect on sigmoid sinus position is inconsistent across populations [14, 24, 26–28]. The variability reported in cadaveric and radiologic studies suggests that sex-related differences are likely secondary to complex interactions between cranial growth, pneumatization, and individual anatomical variation rather than a direct determinant of sigmoid sinus configuration [28, 29].

This study has several notable strengths. First, it addresses a clinically relevant anatomical issue, as variations in sigmoid sinus position may influence surgical corridors and procedural safety in otologic and lateral skull base interventions. Second, the inclusion of a relatively large cohort with a wide adult age range (18–92 years) which provided meaningful exploration of age-related patterns in sigmoid sinus configuration. Third, the use of a surgical reference–based classification system grounded in reproducible anatomical landmarks facilitates standardized reporting and enhances communication between radiologists and surgeons. Compared with earlier cadaveric or pneumatization-based classifications, the reference-line–based system used in this study provides a reproducible, CT-oriented framework that relies on clearly identifiable anatomical landmarks. Its focus on surgical accessibility and bilateral applicability makes it particularly suitable for radiologic evaluation and standardized reporting. Finally, the bilateral evaluation of sigmoid sinus morphology within the same individuals represents an important methodological advantage, allowing assessment of side-to-side variation while minimizing inter-individual confounding when appropriate paired analytical approaches are applied.

Several limitations should be acknowledged. First, the retrospective design limits control over imaging parameters and precludes assessment of causal relationships. Second, mastoid pneumatization was not quantitatively assessed, which limits mechanistic interpretation of the observed associations between sigmoid sinus type, age, and morphometric parameters. In addition, although no cases with documented chronic middle ear disease were identified based on systematic review of clinical records and ICD codes, the possibility of subclinical or undiagnosed conditions cannot be entirely excluded due to the retrospective design. Therefore, the findings should be interpreted as reflecting anatomical variation within the limits of available clinical documentation rather than disease-specific remodeling. Third, surgical outcomes were not evaluated, and thus the clinical implications regarding operative risk remain inferential rather than outcome-based. The study was conducted in a single population, which may limit generalizability to other ethnic or demographic groups. Another limitation of this study is that image evaluations were performed by radiology-trained observers, and no otologist or otologic surgeon participated in the assessment. Although the applied classification system is imaging-based and designed to be reproducible on CT, the absence of direct otologic input may limit interpretation from a strictly surgical perspective. Future studies incorporating joint evaluation by radiologists and otologic surgeons may further enhance clinical applicability and interdisciplinary validity. Because this was a retrospective study based on CT scans obtained for heterogeneous clinical indications, selection bias cannot be excluded, although cases with prior surgery or destructive pathology were excluded and analyses focused on stable osseous anatomy. Finally, because right and left measurements originate from the same individual, within-subject correlation was considered by performing paired analyses and by analyzing each side separately. In addition, although statistically significant associations were identified, the effect sizes were generally small to moderate, and the clinical relevance of these differences remains uncertain. Therefore, the findings should be interpreted primarily as descriptive and hypothesis-generating rather than directly translatable to surgical decision-making. More advanced clustered modeling approaches (e.g., generalized estimating equations or mixed-effects models) were not applied, as the analyses were exploratory in nature; this is acknowledged as a limitation of the study.

## Conclusion

This study aimed to evaluate a clinically oriented classification of sigmoid sinus positioning. Unlike other studies, we did not observe any Type 1 variants. Classifying the sigmoid sinus relative to anatomical reference lines may provide a structured framework for radiologic assessment; however, its role in guiding surgical approaches requires further investigation. These findings contribute to the existing knowledge on sigmoid sinus anatomy.

## Data Availability

No datasets were generated or analysed during the current study.
